# X-ray mirrors with sub-nanometre figure errors obtained by differential deposition of thin WSi_2_ films

**DOI:** 10.1107/S1600577523003697

**Published:** 2023-05-30

**Authors:** Patrice Bras, Christian Morawe, Sylvain Labouré, François Perrin, Amparo Vivo, Raymond Barrett

**Affiliations:** a X-ray Optics Group, ESRF – The European Synchrotron, 71 Avenue des Martyrs, 38043 Grenoble, France; Bhabha Atomic Research Centre, India

**Keywords:** X-ray optics, figure correction, differential deposition, surface roughness, film stress, off-line metrology, Fizeau stitching, long trace profiler, magnetron sputtering

## Abstract

Differential deposition by DC magnetron sputtering was applied to correct for figure errors of X-ray mirrors to be deployed on low-emittance synchrotron beamlines. During the deposition process, the mirrors were moved in front of a beam-defining aperture and the required velocity profile was calculated using a deconvolution algorithm. The surface figure was characterized using conventional off-line visible-light metrology instrumentation (long trace profiler and Fizeau interferometer) before and after the deposition. WSi_2_ was revealed to be a promising candidate material since it conserves the initial substrate surface roughness and limits the film stress to acceptable levels. On a 300 mm-long flat Si mirror the average height errors were reduced by a factor of 20 down to 0.2 nm root mean square. This result shows the suitability of WSi_2_ for differential deposition. Potential promising applications include the upgrade of affordable, average-quality substrates to the standards of modern synchrotron beamlines.

## Abstract 2

The surface figure of an X-ray mirror has been corrected by differential deposition of WSi_2_ films.

## 1. Introduction

As a consequence of recent brilliance improvements on synchrotron sources such as the ESRF Extremely Brilliant Source (EBS) (Raimondi, 2016 ▸), the surface quality requirements for reflective beamline optics have become increasingly stringent. Height errors of X-ray mirror surfaces, for instance, must be limited to the nanometre level over lengths of tens of centimetres whilst maintaining the low surface roughness obtained by the preceding polishing process.

Appropriate surface finishing techniques increasingly rely on deterministic processes which fall into two categories: (1) subtractive methods, where excess material is removed locally – this is the basis of elastic emission machining (Yamauchi *et al.*, 2002 ▸) or ion-beam figuring (Arnold *et al.*, 2010 ▸; Wang *et al.*, 2019 ▸) for instance; (2) additive methods, whereby a corrective thin film of variable thickness is deposited in order to compensate for height variations. This second approach, notably including differential deposition (DD) techniques, has been successfully applied in several research fields (Kilaru *et al.*, 2011 ▸), including X-ray optics (Handa *et al.*, 2008 ▸; Alcock & Cockerton, 2010 ▸; Matsuyama *et al.*, 2018 ▸). DD can be implemented using magnetron sputtering, given its high process stability, versatility and uniformity (Bräuer *et al.*, 2010 ▸). The DD process developed at the ESRF relies on a controlled-speed substrate motion in front of a static sputtering source (Morawe & Peffen, 2009 ▸). All such deterministic processes depend upon accurate measurement of the topography of the optical surface with a sufficient spatial resolution to guide the correction sequence.

The performance of reflective X-ray optics, in particular multilayer (ML) coated mirrors, depends strongly on the quality of the underlying substrates (Morawe *et al.*, 2011 ▸). Consequently, one of the challenges associated with DD is to preserve or even improve initial substrate surface roughness. The roughness of metals tends to increase with layer thickness, which was already observed for DD using Cr (Morawe *et al.*, 2019 ▸). An ML structure involving a metal and C spacers can be successfully used to limit roughness build-up with thickness (Morawe *et al.*, 2021 ▸). Another approach is to use non-metallic compounds such as WSi_2_, which exhibits interesting properties for DD applications, including potential smoothing and limited intrinsic stress (Zhou *et al.*, 2010 ▸).

## 2. Methods and experimental techniques

### 2.1. Thin films and differential deposition

All coatings were made at the ESRF multilayer deposition facility (Morawe & Borel, 2007 ▸) using DC magnetron sputtering. The deposition chamber base pressure was of the order of 10^−5^ Pa. A combination of flow-controlled Ar injection and variable throttle valves with a feedback loop was used to control the process pressure. WSi_2_ films were deposited using a compound target, located at 92 mm from the sample, at a process pressure of 0.1 Pa. The sputtering power was set to 200 W, corresponding to a power density of 0.9 W cm^−2^. The resulting deposition rate maximum, corresponding to the centre of the particle flux, was 0.32 nm s^−1^. Two deposition modes are available – so-called static and dynamic deposition.

Static coatings are performed without substrate motion to characterize the sputtering flux. Laterally, the sample can be mounted with an accuracy of 0.1–0.2 mm with respect to the cathode position. In this case, film thickness is controlled by mechanical shutters.

Dynamic mode involves substrate motion and is used for DD. The approach implemented at the ESRF multilayer laboratory is based on continuous substrate motion at controlled speed in front of the sputtering cathode.

The following equation can be used to describe the thickness distribution *t*(*x*_s_) of a film deposited on a substrate moving at a speed *v*(*x*_m_) in front of a particle source (Morawe & Peffen, 2009 ▸),

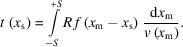


*x*_s_ refers to position in substrate coordinates while *x*_m_ is the motor driver position. *f*(*x*_m_ − *x*_s_) is the normalized, steady-state thickness distribution produced by the particle source at the substrate surface. *R* is the deposition rate at the centre of the distribution. 2*S* corresponds to the full extent of the substrate motion.

While direct film thickness calculation for a given speed profile appears straightforward, the opposite requires a deconvolution process. Based on subroutines initially developed by NASA for deconvolution of astronomical images (Varosi, 1992 ▸), an algorithm based on matrix inversion was developed to solve the problem.

*R* and *f* can be measured on a sample obtained in static deposition and used as input during optimization.

Further details about the procedure are given by Morawe *et al.* (2019 ▸). Repeated duty cycles (back and forth motions) can be applied to obtain the required thickness profile, which better distributes the thermal load on the substrate and allows for mechanically convenient velocity values. Under the given conditions, the substrate temperature does not exceed 100°C.

### 2.2. X-ray reflectivity (XRR) and X-ray diffraction (XRD)

All coatings were characterized on a laboratory X-ray reflectometer with a microfocus Cu tube at 8048 eV (Morawe *et al.*, 2018 ▸). The instrument can be operated with high flux and medium resolution with a Montel-type ML collimator, or in high-resolution mode by inserting an Si(111) double-crystal monochromator further downstream. Specular reflectivity data can be taken with a dynamic range of up to 10^7^. The positioning accuracy of the sample on the instrument is of the order of 0.1 mm. The reflectometer is used together with in-house-developed X-ray reflectivity simulation software, based on the Parratt formalism (Parratt, 1954 ▸), allowing precise determination of thin film thickness, mass density and surface roughness. XRD was performed in high flux mode to maximize the count rate, and the scans were carried out in the θ:2θ geometry.

### 2.3. Long trace profiler (LTP)

The height errors of the surface before and after each correction cycle were determined using the ESRF LTP (Rommeveaux *et al.*, 2008 ▸). This is an in-house-developed deflectometer operating at 633 nm used to perform meridional measurements on planar or non-planar surfaces, up to 1.4 m long, with an accuracy of 0.1 µrad and a lateral resolution of 2 mm. Based on the pencil beam deflectometry technique, an elementary scan yields the slope variation along a single line. The mirror coordinate system reference with respect to the mirror edges is obtained with a precision better than 50 µm using the LTP signal detection in the meridional direction. In the sagittal direction, the alignment accuracy is estimated to be about 0.3 mm and could be improved by using dedicated metrology fiducials on the reflective surface. The mirror is supported by two rods spaced by half of its length in order to analytically subtract gravity effects. The integration of the slope profile allows retrieval of the height profile of the mirror. In the case of a flat, a sphere or a tangential cylinder, the residual shape error profile is then obtained by subtracting a second-order polynomial from the measured height profile. The LTP measurements were used to guide the iterative correction process.

### 2.4. Fizeau interferometer

Fizeau interferometry can provide a two-dimensional map of the surface height topography of the optical surface. The ESRF optical metrology laboratory operates two ZYGO phase-shifting Fizeau interferometers at 633 nm, each with 150 mm-diameter aperture. One is set up in a vertical configuration (GPI-XPZ) allowing measurements of upward reflecting mirrors, and the second in a horizontal configuration (GPI-AT+) for horizontally deflecting mirrors. In order to extend the measurement capability for mirrors up to 1 m long, Fizeau stitching techniques have been developed and implemented (Vivo & Barrett, 2017 ▸; Vivo *et al.*, 2019 ▸) on both instruments. Acquisition of overlapped sub-apertures is ensured with a motorized stitching tool composed of translation, tip-tilt and rotation stages allowing a fine alignment of the mirror while the interferometer stays at a fixed position. The set of sub-apertures is then stitched using *PyLost* (*Python Large Optic Stitching*) software (Adapa, 2020 ▸) to reconstruct the topography of the entire mirror surface. The repeatability achieved is better than 0.05 nm root mean square (RMS) and cross comparison with the LTP has confirmed a sub-nanometre accuracy. In this study the Fizeau interferometric measurements were used to monitor the evolution of the optical surface height errors but was not used as input to guide the correction process.

### 2.5. Stress measurements

The residual stress of the thin films was studied by recording the change of the macroscopic sample curvature and by applying the Stoney equation (Stoney, 1909 ▸). The curvature evolution was measured on thin Si wafer substrates quasi *in situ* using a specific monitor (Morawe *et al.*, 2010 ▸). Coatings during the stress measurements were carried out on 0.405 mm thin Si(100) wafers. The Stoney equation was applied using a Young’s modulus of *E*(Si) = 130 GPa and a Poisson ratio of ν(Si) = 0.28.

## 3. Experimental results

### 3.1. Surface roughness

**Figure 1:**
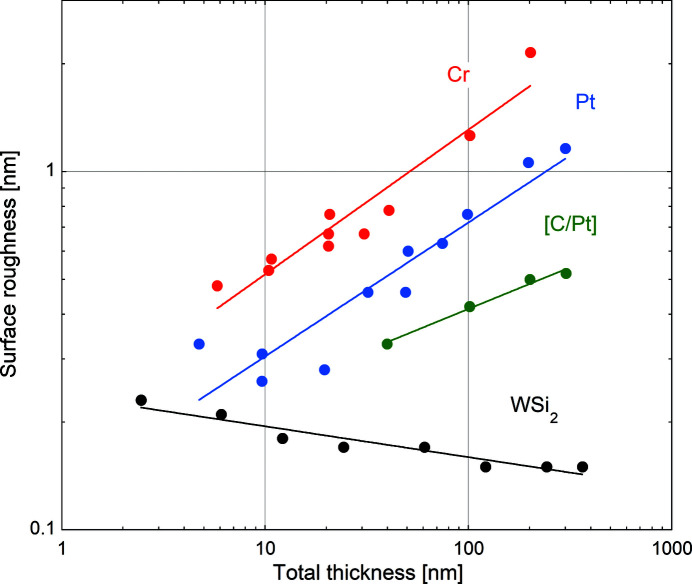
RMS surface roughness of Cr (red), Pt (blue), [C/Pt] (green) and WSi_2_ (black) versus total film thickness. Straight lines correspond to fits using power laws.

The Si wafers used for the roughness studies have a typical surface roughness of 0.3 nm RMS as obtained from simulations to XRR data. Roughness values for single films are also derived from XRR data. Fig. 1 ▸ compares the roughness evolution as a function of film thickness for different material systems. Dots are experimental data and straight lines represent fits using power laws. One observes that the surface roughness of metallic films such as Cr (red dots) and Pt (blue dots) increases with film thickness and values of the order of 1 nm RMS are exceeded above 100 nm film thickness (Morawe *et al.*, 2021 ▸). This effect can be attenuated when carbon spacers are inserted between metallic sublayers as shown for C/Pt (green dots). For WSi_2_ (black dots), the opposite trend is observed and the roughness reduces with increasing film thickness to values below 0.2 nm RMS.

It has been observed that WSi_2_ stays amorphous even at relatively high thicknesses while metals tend to crystallize after a critical thickness that depends on sputtering parameters and growth conditions. WSi_2_ deposition through sputtering was reported to result in a smoother surface as compared with the underlying Si film (Wang *et al.*, 2007 ▸). The main smoothening mechanism invoked relies on impact-induced downhill currents resulting from energetic particle bombardment (Moseler *et al.*, 2005 ▸), which occurs, for instance, during sputter deposition.

**Figure 2:**
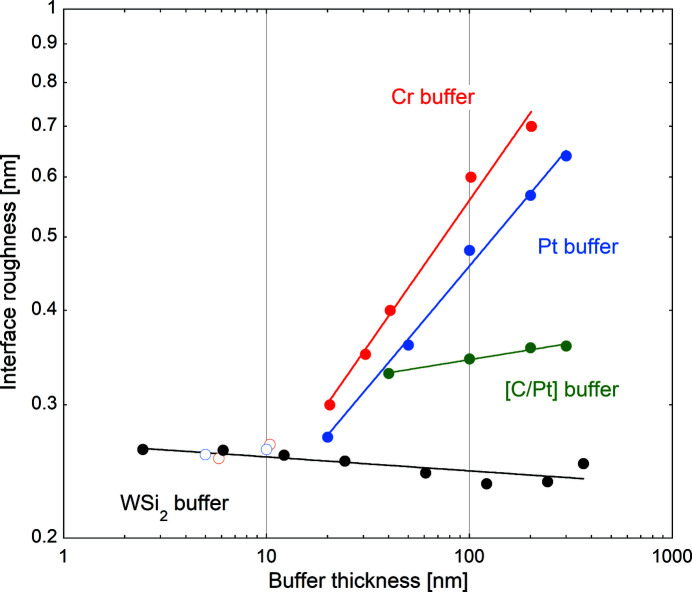
RMS interface roughness of W/B_4_C MLs on buffer layers of Cr (red), Pt (blue), [C/Pt] (green) and WSi_2_ (black) versus buffer layer film thickness. Straight lines correspond to fits using power laws. Open symbols were excluded from the fits.

Single films used to correct for mirror height errors may later act as buffer layers for subsequent ML deposition. In this context, it is important to investigate the impact of the measured surface roughness on the quality of added MLs. Therefore, four series of nearly identical [W/B_4_C]_20_ MLs with a *d*-spacing of 2.5 nm were deposited on top of the single films. Their interfaces are nearly symmetric and the corresponding average roughness versus buffer layer thickness is shown in Fig. 2 ▸. Dots are experimental data and straight lines represent fits using power laws. Open symbols were excluded from the fits. These correspond to samples where the buffer layer roughness was sufficiently low to maintain the ML interface roughness at a constant low level. The overall behaviour is similar to that observed in Fig. 1 ▸ for the underlying single films. The interface roughness strongly increases for Cr (red dots) and Pt (blue dots) buffers thicker than 10 nm to values above 0.6 nm RMS. The slope is less pronounced for C/Pt buffer layers (green dots). The best results are again achieved using WSi_2_ where the ML interface roughness slowly decreases and stays below 0.3 nm. In terms of avoiding roughness increase, this behaviour shows WSi_2_ as being well suited for DD with subsequent ML coatings.

### 3.2. Film stress

**Figure 3:**
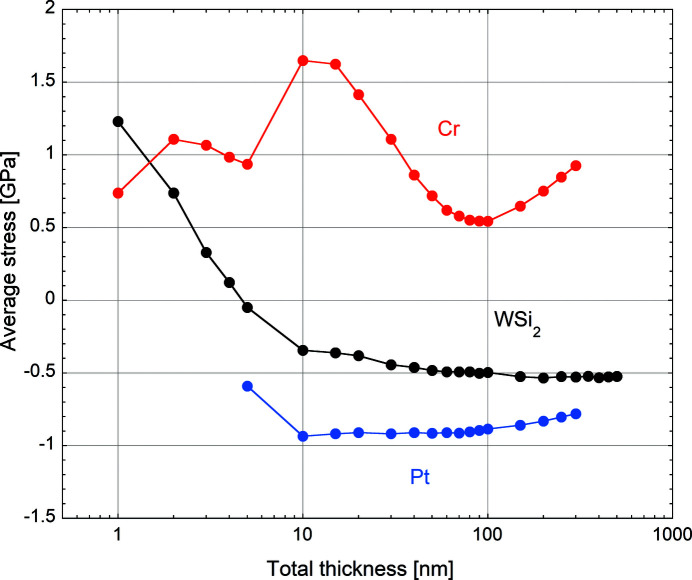
*In situ* average film stress of Cr/Si (red), Pt/Si (blue) and WSi_2_/Si (black) versus thickness. Lines are guides to the eye.

Stress-induced surface curvature change has previously hindered successful DD, especially when Cr was used as a corrective layer (Morawe *et al.*, 2019 ▸). Fig. 3 ▸ shows the evolution of the average stress within thin layers when their thickness increases. Cr films develop tensile stress between 0.5 GPa and 1.7 GPa in the thickness range 1–300 nm. Pt films exhibit compressive stress of similar magnitude with minor variation over the thickness range 5–300 nm. At thickness values below 4 nm, WSi_2_ films exhibit tensile average stress. A transition to compressive stress is observed for higher thicknesses with a magnitude eventually saturating around −0.5 GPa up to a thickness of 500 nm.

A commonly accepted model for thin film growth involves an initial low stress phase characterized by individual islands progressively expanding. Coalescence, which typically occurs at thickness values in the nanometre range, leads to tensile stress resulting from grain boundary formation, as visible for the first nanometres in Fig. 3 ▸. For thicker films, atom incorporation into grain boundaries is believed to create incremental compressive contributions which eventually results in average compressive stress. Additionally, bombardment of energetic species, occurring during magnetron sputtering, tends to enhance compressive stress through ‘atomic peening’ effects (Abadias *et al.*, 2018 ▸). This behaviour is consistent with our observations for WSi_2_.

Relatively low intrinsic stress is an important requirement for differential deposition in order to limit the risk of de­lamination of the corrective layer and minimize changes of the surface curvature. Again, WSi_2_ appears to be a promising candidate material for DD in this respect.

### 3.3. Optimization of deposition parameters

**Figure 4:**
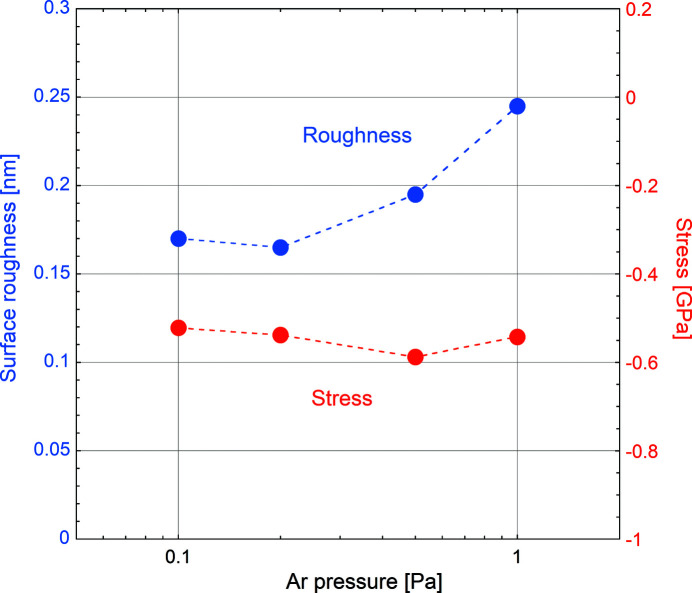
Surface roughness (blue symbols, left ordinate) and stress (red symbols, right ordinate) of 60 nm-thick WSi_2_ films versus Ar pressure. Broken lines are guides to the eye.

The surface roughness and the stress level are important properties to take into account during the optimization of the sputter deposition parameters, in particular the Ar pressure. Fig. 4 ▸ shows both the surface roughness (blue symbols) and the stress (red symbols) of 60 nm-thick WSi_2_ films measured over an Ar pressure range between 0.1 Pa and 1.0 Pa. The surface roughness increases slowly for *p*(Ar) > 0.2 Pa while the (negative) compressive stress shows no significant evolution with increasing Ar pressure. Based on these findings, *p*(Ar) = 0.1 Pa was chosen as the optimum working pressure.

### 3.4. Crystal structure

**Figure 5:**
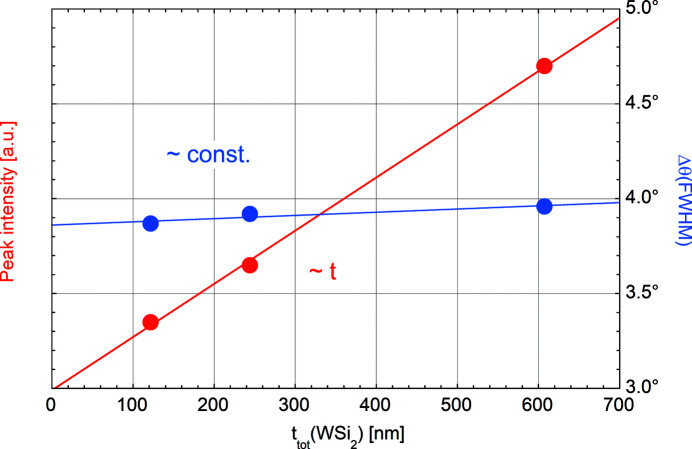
Peak intensity (red dots) and width (blue dots) of the Bragg reflection near θ = 20°. The lines are linear fits to the data.

The crystal structure of the WSi_2_ films was studied using XRD. Apart from the Si(400) substrate reflection, only a weak and broad maximum near θ = 20° was observed. Its position corresponds to the W(110) reflection (θ = 20.18°), which is the most densely packed plane in a body-centred-cubic crystal structure, but would also fit to the WSi_2_(110) peak (Zhang *et al.*, 2020 ▸). Fig. 5 ▸ shows the evolution of the intensity and the full width at half-maximum (FWHM) Δθ of this peak versus film thickness. Its intensity is proportional to the film thickness while its width remains nearly constant. This indicates that the diffraction peak is generated by crystallites of constant size whose number increases proportionally with thickness. Applying the Scherrer equation (Warren, 1990 ▸) to this reflection at θ = 20° returns a vertical particle size of 1.1 nm, which is in good agreement with the results observed by Zhou *et al.* (2010 ▸).

### 3.5. Static flux distribution (SFD)

**Figure 6:**
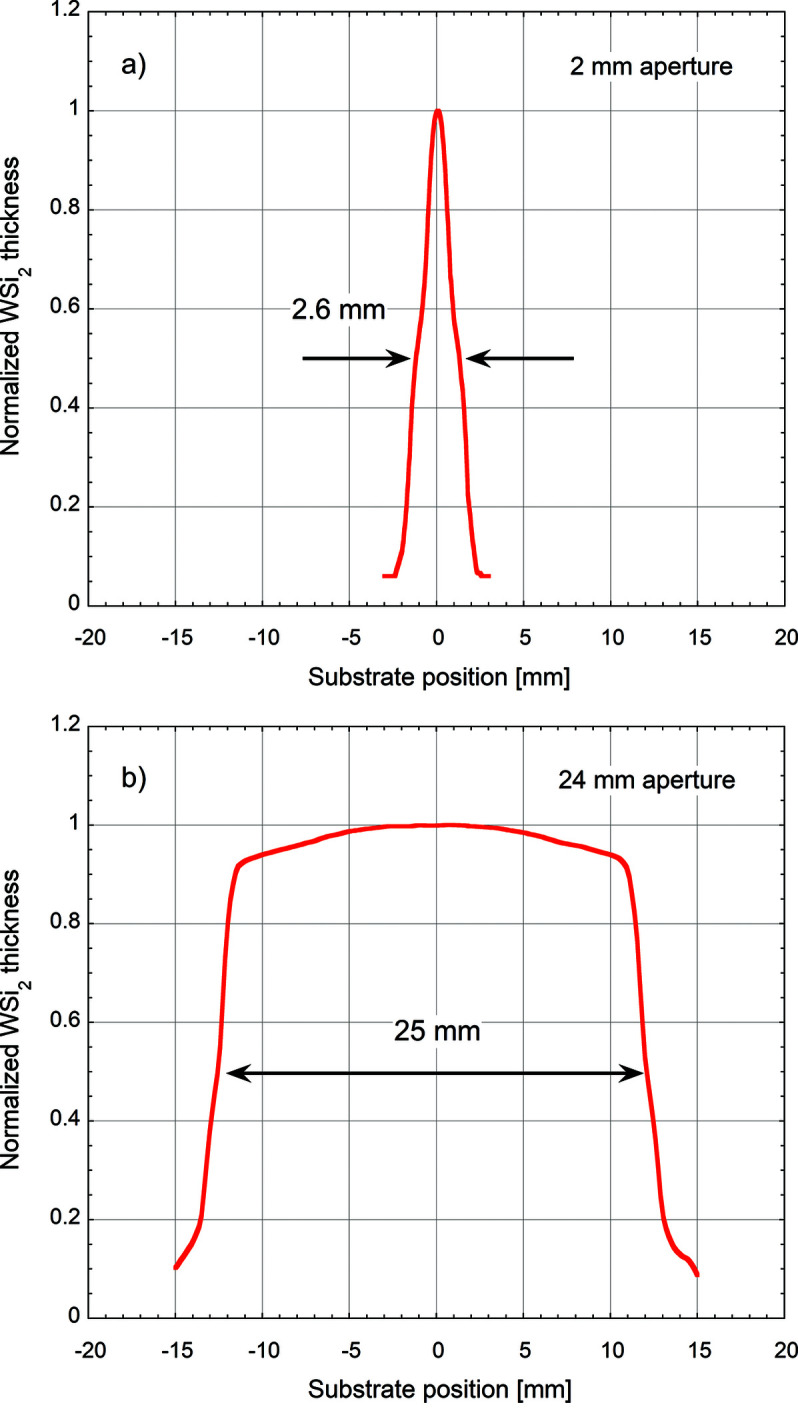
Normalized thickness profiles of WSi_2_ films coated on stationary substrates through apertures of 2 mm (*a*) and 24 mm (*b*).

As a first step, the WSi_2_ particle flux distribution was characterized. WSi_2_ films of thickness 20 nm were deposited on stationary Si wafers through 2 mm- and 24 mm-wide apertures and their local thicknesses were measured with XRR in 0.5 mm and 2 mm steps, respectively. The normalized SFDs are shown in Fig. 6 ▸. The 2 mm aperture [Fig. 6 ▸(*a*)] produces a spike profile with a width of about 2.6 mm (FWHM). The SFD after the 24 mm aperture [Fig. 6 ▸(*b*)] is more rectangular with a FWHM of 25 mm. Both SFDs show two pronounced shoulders, which are images of the two straight erosion lines on the rectangular sputter target that are projected onto the substrate through the beam-defining apertures.

### 3.6. Figure correction of a Si mirror

**Figure 7:**
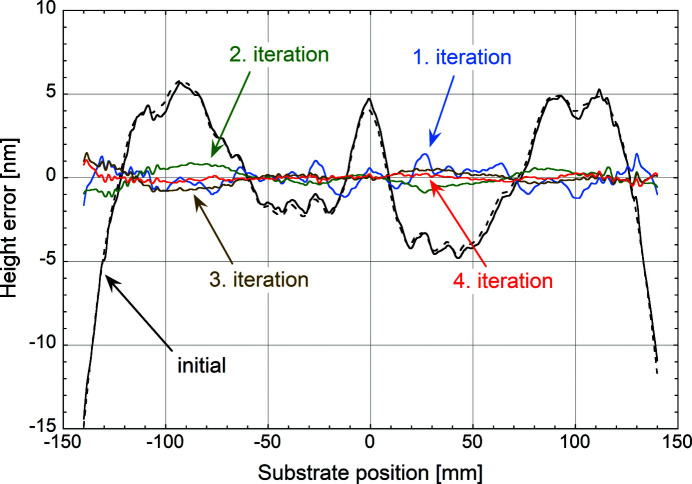
LTP measured surface height errors of the initial Si mirror (black broken line), after an initial uniform layer (black solid line), after the first (blue line), the second (green line) and the third corrective coating (brown line), and after a final uniform layer (red line).

A 300 mm-long, 45 mm-wide and 30 mm-thick Si mirror was selected for correction. Both the surface metrology and the differential deposition technique were applied to the central line along a trace of 280 mm. The initial surface figure was characterized using the LTP. The measured height error profile is displayed in Fig. 7 ▸ (black broken line). To provide identical surface conditions during all subsequent metrology studies, the Si substrate was covered with a 30 nm-thick uniform WSi_2_ film and measured again with the LTP (black solid line in Fig. 7 ▸). Only minor changes of the height errors of the order of 0.1 nm RMS can be detected comparing the two curves. The initial height errors of this mirror were measured to be 3.87 nm (RMS) and 20.1 nm peak to valley (PV). A first corrective WSi_2_ coating with an average thickness of 20 nm was applied. During this process, the 24 mm aperture was inserted into the particle beam and only height errors extending over periods of more than 50 mm were attempted to be removed. The result is shown as the blue curve in Fig. 7 ▸. The long-period variations have essentially disappeared leaving only errors below that length scale. After this first correction, the height errors were reduced to 0.57 nm (RMS) and 3.09 nm (PV). A second corrective coating with an average WSi_2_ thickness of 4 nm was carried out, this time through the 2 mm aperture, with the aim to correct for errors on shorter length scales. The outcome is given as the green curve in Fig. 7 ▸. The short-period errors have been significantly attenuated. However, low-amplitude long-range errors remain, apparently resembling the initial mirror shape. The height errors have been slightly reduced to 0.46 nm (RMS) and 2.03 nm (PV). At this stage, the residual long-period errors dominate the total balance. To remove the remaining height errors, a third corrective WSi_2_ coating with an average thickness of 3 nm was added, this time returning to the 24 mm aperture, since short-period errors have been sufficiently attenuated. The result is added as the brown curve to Fig. 7 ▸. Surprisingly, the error profile between the second and third corrections seems to have flipped over to the opposite direction while maintaining its amplitude. The corresponding errors remain on the same level with 0.39 nm (RMS) and 2.34 nm (PV). It seems highly unlikely that the third WSi_2_ coating caused such an effect, since the achievable thickness control is much better than the observed departure from the expected result.

The origin of this phenomenon is thought to be linked to the partial transparence for visible light of thin WSi_2_ correction layers. This allows interaction of the visible-light beam of the LTP with buried interfaces of the corrected mirror profile. Such interfaces can form by oxidation of the native WSi_2_ surface after removal of the mirror from the vacuum vessel. Parasitic reflections and interference can cause variations in the LTP signal and falsify the results of the metrology measurements. The correlation between the residual errors and the initial mirror shape support this assumption.

To verify the above hypothesis, in a fourth iteration, the mirror was over-coated with a uniform 60 nm-thick WSi_2_ film that, based on experimental measurements, should have a transmittance below 1% for visible light and thus largely attenuate parasitic reflections from buried interfaces. The LTP measurement after this coating is shown in Fig. 7 ▸ as the red curve. The error amplitude compared with the second and third corrections has significantly dropped and no further height variations have been introduced. The measured residual height errors are 0.19 nm (RMS) and 1.58 nm (PV). After removal of edge effects and reduction of the analysis length to 260 mm the errors even drop to 0.14 nm (RMS) and 0.86 nm (PV).

**Figure 8:**
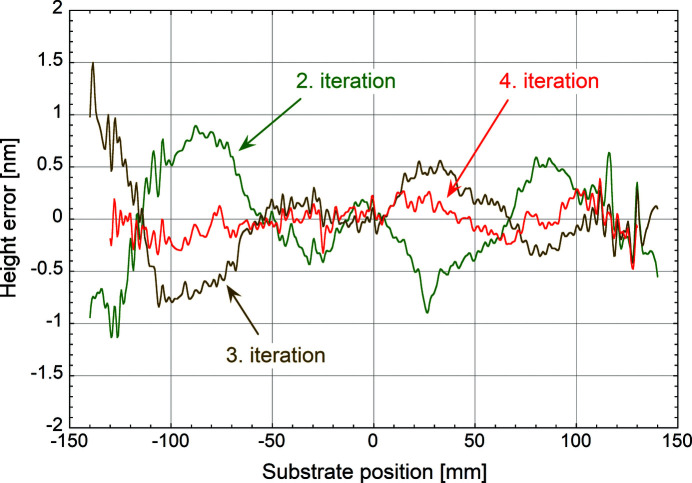
LTP measured surface height errors after the second (green line) and the third corrective coating (brown line), and after a final uniform layer (red line).

For improved visibility, Fig. 8 ▸ shows the curves of the last three iterations only using the same colour code as Fig. 7 ▸. The significant reduction of the measured surface figure after the fourth iteration is evident. At the same time, the principal high-frequency features remain visible in all three measurements, which underlines the quality of the LTP data. It also shows that, as expected, these short spatial-period errors are not corrected with the current protocol.

**Figure 9:**
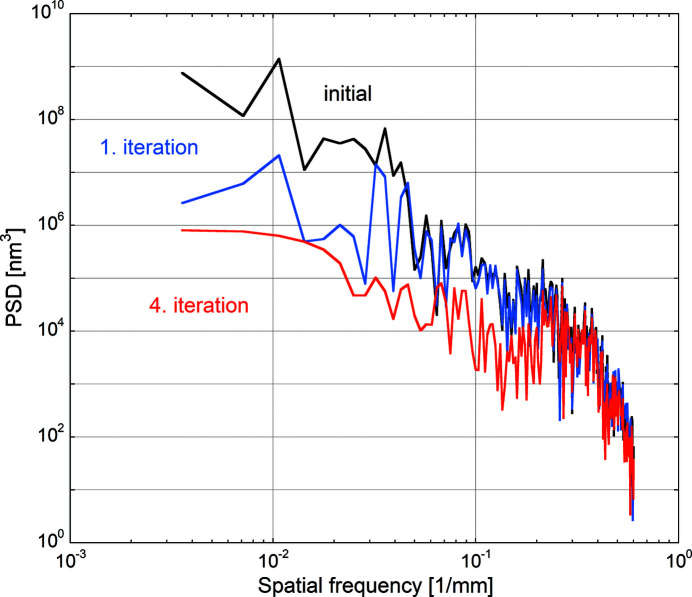
PSD spectra calculated from the LTP profiles for the initial situation (black curve), after the first iteration (blue curve) and after the fourth iteration (red curve).

Another way to analyse the surface figure evolution is to transform the data into frequency space and calculate the corresponding height errors’ power spectral densities (PSDs). The PSDs were calculated using standard routines available in the SciPy module (Virtanen *et al.*, 2020 ▸) applied to the LTP data after integration. A Tukey (tapered cosine) window with a ratio of taper to constant sections of 0.2 and a total width of 280 mm is applied prior to PSD calculation in order to reduce frequency leakage phenomena during the PSD calculation due to the extremities of the measured area (Press *et al.*, 2007 ▸). Figs. 9 ▸ and 10 ▸ present the PSD and the corresponding square root of the cumulative power spectral density [(CPSD)^1/2^] of the height error profiles measured on the initial substrate after uniform WSi_2_ deposition, after the first and after the fourth correction iteration. The CPSD is the integral over frequency of the one-sided line PSD and provides a graphic representation of the surface variance build-up with increasing frequency. The square root of this function represents the RMS height error build-up and shows rather clearly the contribution of different spatial frequencies to the overall shape errors.

Fig. 9 ▸ shows a clear reduction in PSD magnitude in the spatial frequency range below 0.03 mm^−1^ after the first correction, corresponding to the spatial wavelength range above 30 mm. This wavelength range is of the same order as the large aperture width used for initial correction, which confirms the statements made in Section 3.5 and proves its effectiveness in correcting long-wavelength height errors.

Comparing the PSDs after the first (large aperture, blue curve) and fourth (narrow aperture, red curve) correction iteration shows a reduction in the intermediate spatial frequency range between 0.03 and 0.2 mm^−1^. Height error contributions extending over several millimetres were effectively corrected by differential deposition using the narrow aperture.

For higher lateral frequencies, no obvious gain is observed as even the narrow slit is too wide to correct the corresponding surface features.

**Figure 10:**
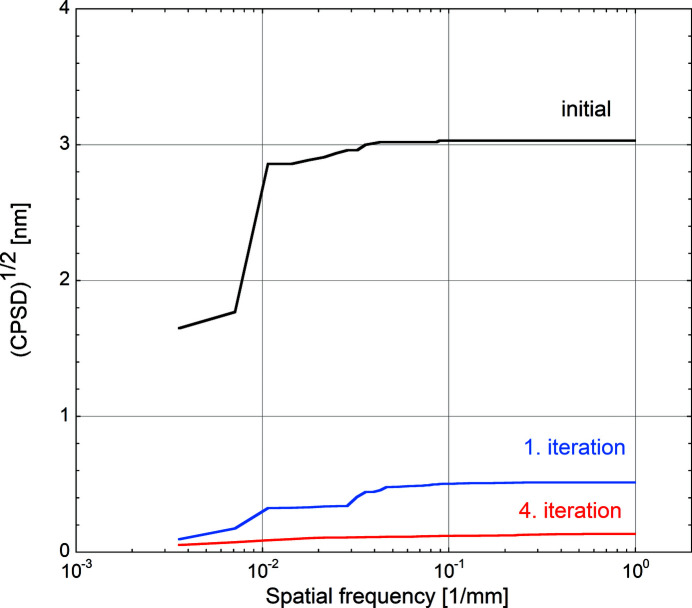
(CPSD)^1/2^ data calcluated from LTP measurements for the initial situation (black curve), after the first iteration (blue curve) and after the fourth iteration (red curve).

The (CPSD)^1/2^ in Fig. 10 ▸, here on a linear scale, enables an integral view on the same phenomenon seen in Fig. 9 ▸, but which reduces the noise level and emphasizes the frequency dependence of the attenuation of the height errors after subsequent figure corrections.

**Figure 11:**
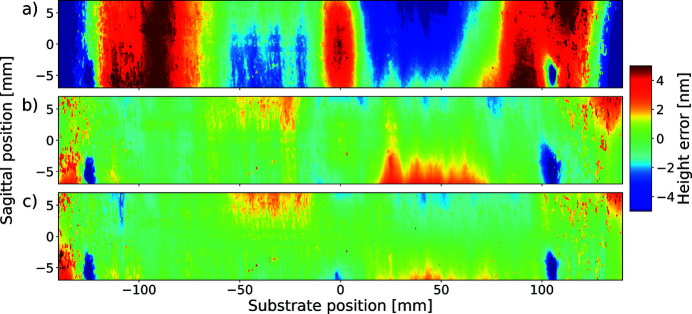
2D surface height error profiles measured with the Fizeau interferometer for the initial situation (*a*), after the first iteration (*b*) and after the fourth iteration (*c*).

Two-dimensional surface height error images, obtained by Fizeau stitching, on the initial surface and after the first and the fourth correction iterations are shown in Fig. 11 ▸. The central profile, corresponding to sagittal position 0 mm, was chosen as the reference for correction. The clear improvement in surface height error profile with successive corrections, already observed in one dimension with the LTP, is also visible in two dimensions.

**Figure 12:**
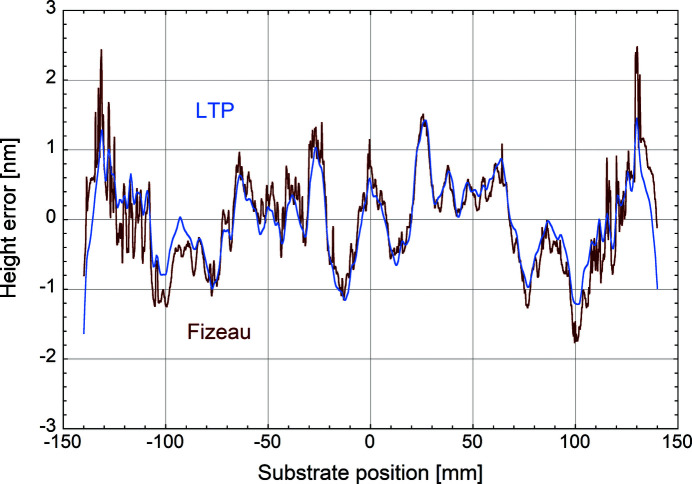
Surface height errors after the first corrective coating measured with the LTP (blue line) and the Fizeau interferometer (brown line).

Fig. 12 ▸ compares the LTP height error data (blue line) after the first correction with the corresponding central profile measured with the Fizeau interferometer (brown line). Apart from the higher spatial resolution obtained from the Fizeau, the agreement between the two instruments is excellent, which underlines the capacity to provide precise metrology feedback during the correction process. Note that, in the present work, all correction steps are based on the respective LTP data sets.

Another important aspect is that the surface profile can vary significantly in the sagittal direction. Sample positioning repeatability during height or slope error measurements between iterations, both along the meridional and the sagittal direction, is of paramount importance for the correction accuracy. Fiducial marks could be used for future mirror corrections by differential deposition to improve the positioning capabilities.

## 4. Discussion

**Figure 13:**
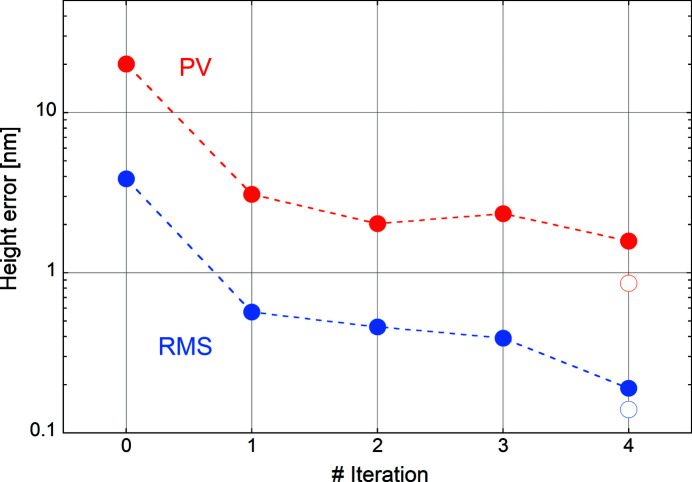
RMS (blue solid circles) and PV (red solid circles) height errors after subsequent iterations measured over a length of 280 mm. Open circles indicate the respective values over a length of 260 mm. Broken lines are guides to the eye.

The evolution of the height errors after subsequent iterations is summarized in Fig. 13 ▸ on a logarithmic scale. Solid circles indicate the RMS (blue) and the PV (red) errors measured over a length of 280 mm. The open circles after the fourth iteration show the respective values taken over a reduced analysis length of 260 mm. The moderate gain between iterations 1 and 2 is caused by the correction of shorter spatial periods that have less impact on the global figure error of the mirror. Visible-light transmittance measurements on WSi_2_ thin films coated on glass show that we expect more than 50% transmission for a thin film of a few nanometres. The average thickness of the third iteration corrective coating is 3 nm. The stagnation between iterations 2 and 3 is then probably linked to the transparency of the correction layer and the presence of buried interfaces, which perturb the metrology data. The final gain between iterations 3 and 4 supports this hypothesis since the physical topography of the mirror surface after the deposition of a uniform layer in iteration 4 is expected to be the same as after iteration 3. Since iteration 3 was very likely based on distorted metrology data and led to no further improvement, the application of the thick uniform top layer already after iteration 2 may have returned a comparable final result.

While the differential deposition process has reached a reliable level of accuracy and convergence, the visible-light metrology, though sufficiently precise, is complicated by potential parasitic effects of the transparency of the thin corrective layers and buried interfaces that can form between successive runs. The deposition of thicker layers appears to reduce their impact and should be explored further.

## 5. Summary and outlook

Differential deposition based on DC magnetron sputtered WSi_2_ films combined with off-line visible-light metrology was developed to improve the figure of reflective X-ray optics over length scales between 5 mm and 300 mm. The choice of WSi_2_ enables the deposition of films of low roughness and moderate intrinsic stress over a wide thickness range beyond 100 nm. After three corrective coatings, sub-nanometre height errors were achieved on an Si mirror over a length of 260 mm. The total error reduction was about 20 times. The superior accuracy of visible-light metrology measurements is highly valuable for successful differential deposition. However, reflections at buried interfaces were revealed to complicate the measurement. Possible mitigation strategies involve the deposition of thicker layers to avoid intermediate reflections and corresponding optical measurement disruption. A new compact ML coating system, currently under commissioning at the ESRF, should allow superior accuracy to be reached in terms of substrate motion speed profiles which is beneficial for differential deposition. The use of narrower apertures could also, in principle, extend the correction capabilities to higher spatial frequencies. Differential deposition using WSi_2_ thin films is a promising technique for significantly improving the surface figure errors of X-ray optics. Next steps involve combination of DD with ML deposition and beamline testing to assess the impact of surface correction in an operational environment.
